# EEG Responses to TMS Are Sensitive to Changes in the Perturbation Parameters and Repeatable over Time

**DOI:** 10.1371/journal.pone.0010281

**Published:** 2010-04-22

**Authors:** Silvia Casarotto, Leonor J. Romero Lauro, Valentina Bellina, Adenauer G. Casali, Mario Rosanova, Andrea Pigorini, Stefano Defendi, Maurizio Mariotti, Marcello Massimini

**Affiliations:** Department of Clinical Sciences “L. Sacco”, Università degli Studi di Milano, Milan, Italy; Cuban Neuroscience Center, Cuba

## Abstract

**Background:**

High-density electroencephalography (hd-EEG) combined with transcranial magnetic stimulation (TMS) provides a direct and non-invasive measure of cortical excitability and connectivity in humans and may be employed to track over time pathological alterations, plastic changes and therapy-induced modifications in cortical circuits. However, the diagnostic/monitoring applications of this technique would be limited to the extent that TMS-evoked potentials are either stereotypical (non-sensitive) or random (non-repeatable) responses. Here, we used controlled changes in the stimulation parameters (site, intensity, and angle of stimulation) and repeated longitudinal measurements (same day and one week apart) to evaluate the sensitivity and repeatability of TMS/hd-EEG potentials.

**Methodology/Principal Findings:**

In 10 volunteers, we performed 92 single-subject comparisons to evaluate the similarities/differences between pairs of TMS-evoked potentials recorded in the same/different stimulation conditions. For each pairwise comparison, we used non-parametric statistics to calculate a Divergence Index (DI), i.e., the percentage of samples that differed significantly, considering all scalp locations and the entire post-stimulus period. A receiver operating characteristic analysis showed that it was possible to find an optimal DI threshold of 1.67%, yielding 96.7% overall accuracy of TMS/hd-EEG in detecting whether a change in the perturbation parameters occurred or not.

**Conclusions/Significance:**

These results demonstrate that the EEG responses to TMS essentially reflect deterministic properties of the stimulated neuronal circuits as opposed to stereotypical responses or uncontrolled variability. To the extent that TMS-evoked potentials are sensitive to changes and repeatable over time, they may be employed to detect longitudinal changes in the state of cortical circuits.

## Introduction

Several studies have suggested that the combination of high-density electroencephalography (hd-EEG) and transcranial magnetic stimulation (TMS) [1], [2] may be employed to directly and non-invasively gauge cortical excitability and connectivity in humans [3]–[16]. Global and/or local changes in the excitability and connectivity patterns of cortical circuits underlie most neuropsychiatric conditions and their treatment. Thus, at least in principle, TMS/hd-EEG may be employed at the patient's bedside to track over time pathological alterations, plastic changes and therapy-induced modifications in cortical circuits.

A first step to evaluate the potential of TMS/hd-EEG as a diagnostic and monitoring tool involves defining the sensitivity and repeatability of this technique. In other words, before employing TMS/hd-EEG at the patient's bedside, one would like to assess to what extent TMS-evoked potentials reflect particular electrophysiological properties of the stimulated cortical circuits rather than a stereotypical brain's reaction, or uncontrollable variability. Indeed, TMS-evoked potentials would have limited diagnostic/monitoring application if they were found not to change when different neuronal subsets are stimulated, or if they were found to vary randomly when stimulation parameters are kept constant. Ideally, TMS-evoked potentials, recorded across different sessions in a healthy brain, should always change significantly if stimulation parameters are varied (100% *sensitivity*) and should not change if stimulation parameters are kept constant (100% *repeatability*).

Separate experimental evidences have suggested that TMS-evoked potentials have a certain degree of sensitivity to changes in stimulation parameters, such as location [10], [12], [17], [18], intensity [13], [19] and direction of the induced current with respect to the cortical surface [15]. Moreover, a few works have demonstrated that TMS-evoked potentials can also detect changes in the state of cortical circuits, such as the ones induced by alcohol intake [9], [11], [20], by falling asleep [16], [21] and by induction of cortical potentiation with repetitive TMS [22]. To the best of our knowledge, repeatability has been, so far, evaluated only in one work by Lioumis *et al.* [23]. This work suggested that, at least at the group level, the amplitude and latency of selected components of TMS-evoked potentials tend to be stable over time when stimulation parameters are constant.

Altogether, the evidence reported above, although not systematic, suggest that TMS/hd-EEG is a reliable technique. The aim of the present work is to perform a statistical joint evaluation of the sensitivity and repeatability of TMS/hd-EEG measures. Overall, we recorded 100 TMS/hd-EEG sessions in 10 healthy volunteers and we systematically performed 92 pairwise comparisons at the single-subject level between the TMS-evoked potentials obtained either using different stimulation parameters, namely site, intensity, and angle of stimulation (change comparison - C), or keeping stimulation parameters constant over time (no-change comparison - NC). For each comparison, we applied non-parametric statistics to compute a Divergence Index (DI), i.e. the percentage of spatial-temporal samples that differed significantly between two sessions of TMS-evoked potentials. At this point, considering each DI as a threshold, we performed receiver operating characteristic (ROC) analysis and we computed the true positive rate (the fraction of C comparisons with DI > threshold) and the true negative rate (the fraction of NC comparisons with a DI < threshold).

ROC analysis showed that the overall accuracy of TMS/hd-EEG in disclosing changes in the stimulation parameters was 96.7%, with 95% sensitivity and 100% specificity (i.e. repeatability) at a DI threshold of 1.67%. These results demonstrate that TMS-evoked potentials are sensitive (non-stereotypical) and repeatable (non-random) responses and that they reflect, deterministically, particular properties of the stimulated set of cortical neurons. To the extent that TMS-evoked potentials are sensitive and repeatable for changes in the stimulation parameters, they may also be accurate in detecting longitudinal changes in the state of cortical circuits.

## Materials and Methods

### Participants

Ten right handed healthy volunteers (7 males, 3 females, mean age 26.9 years) were enrolled into the study after a neurological screening to exclude potential adverse effects of TMS. Subjects with medical history of seizures, convulsions, loss of consciousness and traumatic brain injury, carriers of intracranial metallic objects and/or of cardiac pace-makers were excluded from the study. The entire experimental procedure was approved by the Local Ethical Committee of the Hospital “L. Sacco” and each volunteer gave written informed consent to participation.

### TMS targeting

Structural magnetic resonance images (MRI) were recorded from all subjects at 1 mm^3^ spatial resolution (1T Philips scanner). Three TMS targets were identified on individual MRIs in the left occipital lobe (Brodmann's area - BA19), the left parietal lobe (BA7) and the left frontal lobe (BA6). Precision and reproducibility of stimulation were achieved by using a Navigated Brain Stimulation (NBS) system (Nexstim Ltd., Helsinki, Finland), that employs a 3D infrared Tracking Position Sensor Unit to map the positions of TMS coil and subject's head within the reference space of individual structural MRI. In addition, the NBS system computes on-line the distribution and intensity (V/m) of the intracranial induced electric field using a locally best-fitting spherical model of the subjects' head and brain and taking into account the exact shape, 3D position and orientation of the TMS coil. Stimulation intensity, expressed as a percentage of the maximal output of the stimulator, was kept between 40–75% for all subjects, corresponding to an electric field between 110–120 V/m on the cortical surface. In each area, the TMS hot spot (i.e. location of the maximum electric field induced by TMS on the cortical surface) was always kept on the convexity of the gyrus, about 1 cm lateral to the midline. These medial stimulation sites were selected because they are easily accessible and far from major head or facial muscles whose unwanted activation may affect EEG recordings. The reproducibility of the stimulation coordinates across sessions was guaranteed by a virtual aiming device that indicated in real-time any deviation from the desired target greater than 3 mm. The TMS stimulator consisted of a Focal Bipulse 8-Coil (mean/outer winding diameter ca. 50/70 mm, biphasic pulse shape, pulse length ca. 280 µs, focal area of the stimulation hot spot 0.68 cm^2^) driven by a Mobile Stimulator Unit (Eximia TMS Stimulator, Nexstim Ltd., Helsinki, Finland). The coil was always placed tangentially to the scalp, in order to optimize transmission of the magnetic field to the cortical surface. TMS pulses were delivered at an inter-stimulus interval randomly jittered between 700–900 ms (equivalent to ca. 1.1–1.4 Hz).

### EEG recording

Continuous hd-EEG was recorded using a 60-channel TMS-compatible amplifier (Nexstim Ltd., Helsinki, Finland). This equipment ensured artefact-free EEG recordings starting from 8 ms after the TMS pulse [1]. Impedance at all electrodes was kept below 5 kΩ. EEG signals were band-pass filtered between 0.1–500 Hz and sampled at 1,450 Hz with 16 bit resolution. Vertical electrooculogram (EOG) was recorded by two extra sensors. A total of about 200 trials were collected for each TMS/hd-EEG session. Contamination of TMS-evoked potentials by auditory responses to the clicks produced by the TMS coil's discharge was prevented by masking noise and by placing a thin layer of foam between coil and scalp. After each session, electrodes' position was digitized using a 3D Infrared Tracking Position Sensor Unit (for more details about the EEG recording procedures see [16], [21], [24]).

### General experimental design

The experimental protocol consisted of two main arms, aimed at evaluating the sensitivity and the repeatability, respectively, of TMS-evoked potentials. In order to test for sensitivity, different, randomly ordered, TMS sessions were performed in the same day (day1) varying only one stimulation parameter at a time (either site, or intensity, or angle of the TMS-induced current). TMS-evoked potentials were considered sensitive to the extent that they changed when stimulation parameters were changed. In order to evaluate repeatability, a subset of TMS sessions was repeated later in the same day (day1) as well as one week afterward (day8), without changing any stimulation parameter. TMS-evoked potentials were considered repeatable to the extent that they did not change over time when stimulation parameters were kept constant. In order to quantify sensitivity, single-subject pairwise comparisons were performed between TMS-evoked potentials obtained with different stimulation parameters. We called these comparisons “change comparisons” (C). To quantify repeatability, we performed single-subject pairwise comparisons between TMS-evoked potentials with identical stimulation parameters. We called these comparisons “no change comparisons” (NC). As described below, each pairwise comparison involved applying a non-parametric test based on random permutations between the single-trial TMS-evoked potentials of the two sessions.

### Change comparison (sensitivity)

#### Stimulation site

Pairwise comparisons were carried out between TMS-evoked responses to perturbation of BA6, BA7, and BA19 for each subject separately. Using the NBS, stimulation intensity (I%, expressed as a percentage of the maximum stimulator's output) was always adjusted in each subject and in each area in order to compensate for local differences in scalp-to-cortex distance and to generate a comparable electric field between 110–120 V/m. The NBS was also used to keep the angle of stimulation parallel to the cortex midline (0° angle). In this case, the total number of pairwise comparisons was 22 instead of 30, because in 4 out of 10 subjects cortical responses to perturbation of one area (BA6 in 3 subjects and BA19 in 1 subject) were corrupted by artefacts and therefore excluded from the analysis.

#### Stimulation intensity

Single-subject pairwise comparisons were carried out between TMS-evoked responses obtained at I% and I%+10%. Using the NBS, the stimulation target, as well as the angle of the induced currents were kept unvaried. A total of 20 comparisons were performed, involving BA6 in 4 subjects, BA7 in 7 subjects, and BA19 in 9 subjects.

#### Stimulation angle

Pairwise comparisons were carried out between TMS-evoked responses obtained with a stimulation angle of 0° and responses obtained after clockwise rotating the angle by 45° and 90°. Intensity and site of stimulation were kept constant. Overall, 20 comparisons were evaluated, involving BA6 in 2 subjects, BA7 in 4 subject, and BA19 in 4 subjects (10 comparisons 0° vs. 45° and 10 comparisons 0° vs. 90°).

### No change comparison (repeatability)

#### Same day

For each subject, the first TMS session (intensity I%, direction 0°, stimulation site either BA6, or BA7, or BA19) was compared with an identical session repeated on day1, at the end of the experiment, without changing any stimulation parameter (10 comparisons). This procedure allowed to control for possible plasticity-related modifications induced by repeated TMS sessions.

#### One week apart

A subset of the TMS/hd-EEG sessions recorded on day1 (intensity I% and direction 0°) was compared with identical sessions accurately replicated on day8, namely stimulation of BA6 in 4 subjects, of BA7 in 7 subjects and of BA19 in 9 subjects. In these cases, we carefully controlled that not only the stimulation parameters, but also other environmental and subjective conditions (such as daytime, room brightness, subject's vigilance level) were exactly the same. To further reduce sources of measurement variability, electrodes digitization was used to ensure that the relative position between the EEG cap and subject's head did not differ across the two sessions.

### Data Analysis

#### EEG pre-processing

Data analysis was carried out using MATLAB® (2006a, The MathWorks, Natick, MA). Visual inspection of single-trial recordings was performed by a trained experimenter after automatic rejection of trials with EOG >70 µV and/or with absolute power of EEG channel F8 in the fast beta range (>25 Hz) exceeding 0.9 µV^2^/Hz [25], most likely contaminated by ocular and/or muscular activity. TMS-evoked potentials were computed by averaging a minimum of 150 selected artefact-free single trials, in order to obtain a good signal-to-noise ratio. Subsequently, channels residually affected by large artefacts or with poor signal-to-noise ratio were excluded from further analysis. Finally, the average responses were band-pass filtered (2–80 Hz), downsampled to 725 Hz, and re-referenced to the common average reference.

#### Statistical analysis

We implemented a non-parametric permutation-based statistical procedure to perform pairwise comparisons between TMS-evoked potentials, and to synthesize their degree of diversity in a single value (divergence index - DI), corrected for multiple hypothesis testing.

**Figure 1:**
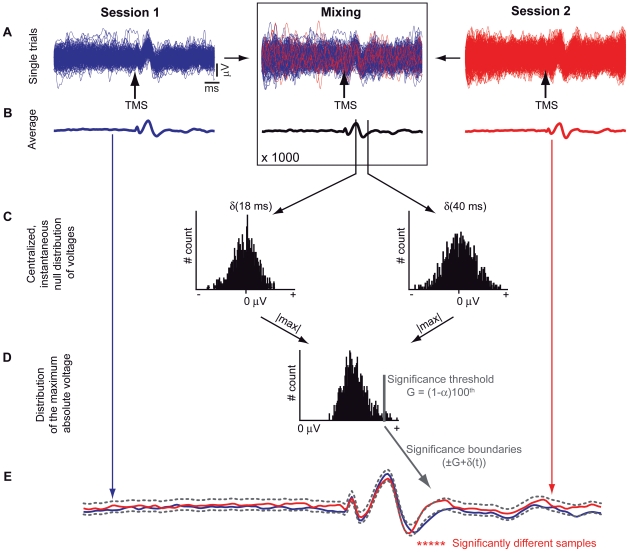
Non-parametric statistical procedure to perform single-subject pairwise comparisons between TMS-evoked potentials. Single-trial recordings from two different conditions (blue and red lines) were randomly mixed 1000 times (A) and averaged (B). Instantaneous distributions of averaged voltages were computed and centralized around zero by keeping record of the displacement δ(t) (C). The distribution of maximum absolute values of each centralized distribution was computed and used to define a significance threshold G as the (1-α)100^th^ percentile (D). Significance boundaries (gray dotted lines) were computed as (±G + δ(t)) and used to define the significantly different time samples (red stars) between conditions at a specific channel (E).

At first, a Wilcoxon rank-sum test was applied to check that the baselines (250 ms pre-stimulus) of the single trials, contributing to the two TMS-evoked potentials to be compared, had the same distribution. In case of a negative result, the most deviated trials were removed and the test was repeated until the baseline distributions of the two groups of trials were statistically equivalent (*P*>0.05). The percentage of rejected trials was always less than 5%. At this point, we could test the null hypothesis that two sets of TMS-evoked potentials (each one composed by 60 EEG channels by 182 time points, corresponding to 250 ms post-stimulus sampled at 725 Hz) are equivalent. If this is the case, “mixing” together, in any random combination, the single trials collected during the two TMS/hd-EEG sessions should always result in statistically equivalent TMS-evoked potentials. Otherwise, the null hypothesis can be rejected. Thus, for each comparison, 1000 “mixed” TMS-evoked potentials were obtained by randomly mixing and averaging 1000 times the single trials collected in two different sessions (Fig. 1A,B). The set of 1000 values at each post-stimulus time sample represented the instantaneous empirical null probabilistic distribution of the voltage of the TMS-evoked potentials. In order to correct for multiple comparisons in time, we computed a single distribution for the whole time interval as follows: i) all instantaneous distributions were centralized around zero, by shifting them by an amount δ(t) (Fig. 1C); ii) for each centralized distribution, we computed the maximum absolute value (Fig. 1D); iii) the one-tail (1-α)100^th^ percentile of the distribution of the maximum absolute values was used to estimate a significance threshold G for the whole time window of interest (Fig. 1D); iv) two boundaries were computed as (+G+δ(t)) and (–G+δ(t)). The temporal profile of these boundaries is modulated by δ(t), since G is a fixed threshold. The null hypothesis of equivalence between two TMS-evoked responses at each time sample t was rejected with probability of false positives α corrected for multiple comparisons when at least one of the two original potentials at that time sample lay beyond the significance boundaries (Fig. 1E). Finally, for each comparison the DI was defined as the percentage of significantly different time samples in the first 250 ms post-stimulus in all 60 EEG channels out of the total number of spatial-temporal samples. In this way, the DI was systematically calculated at the sensor level for all pairwise comparisons (n = 92). As a proof of concept, the same statistical procedure was also carried out at the source level on the time series of regional cortical currents in one subject (see *Source modeling* paragraph below). Cortical meshes were automatically parcellated into subregions (Automated Anatomical Lobules classification) using the masks provided by WFUPickAltas tool (freely available at: http://www.ansir.wfubmc.edu; [24], [26], [27]).

#### Source modeling

The Statistical Parametric Mapping software package (SPM5, freely available at http://www.fil.ion.bpmf.ac.uk/spm) was used to warp individual MRIs to the Montreal Neurological Institute atlas and to compute cortical meshes (7204 vertices each). EEG sensors and individual meshes were co-registered by rigid rotations and translations of anatomical landmarks (nasion, left and right tragus). Conductive head volume was modeled according to the 3-spheres BERG method [28] as implemented in the Brainstorm software package (freely available at http://neuroimage.usc.edu/brainstorm). Finally, inverse solution was computed on each single trial by applying the empirical Bayesian approach [24], [29]–[33].

#### Receiver Operating Characteristic (ROC) analysis

The statistical analysis described above, yielded 92 DIs, resulting from 62 C comparisons (stimulation site, intensity, angle) and from 30 NC comparisons (same day, one week apart). ROC analysis was applied to evaluate the overall ability of TMS-evoked potentials in disclosing well-controlled modifications of stimulation parameters against measurement variability/error. Briefly, each measured DI was set as threshold to decide whether a change occurred (> threshold), or not (< threshold). Thus, the true positive rate (sensitivity%) and the true negative rate (specificity, or repeatability%) was computed for all 92 DI thresholds. Then, we plotted the ROC curve as sensitivity% vs 100-specificity% using a Matlab script (freely available at http://www.mathworks.com/matlabcentral/fileexchange/19950; Cardillo G., 2008: *ROC curve: compute a Receiver Operating Characteristics curve*). The optimal DI threshold was set in correspondence to the maximum of the Younden index [34], computed as [sensitivity + specificity - 1]. The percentage of correct classifications across all pairwise comparisons was measured to quantify accuracy of TMS/hd-EEG, while the area under the ROC curve yielded the probability of ranking the DI of a randomly chosen C comparison higher than the DI of a randomly chosen NC comparison.

## Results

Starting from 100 TMS/hd-EEG sessions recorded in 10 healthy subjects, we performed 62 C comparisons (22 for changes in stimulation site, 20 in stimulation intensity and 20 in stimulation angle) and 30 NC comparisons (10 same-day and 20 one-week-apart recordings with the same stimulation parameters).

**Figure 2:**
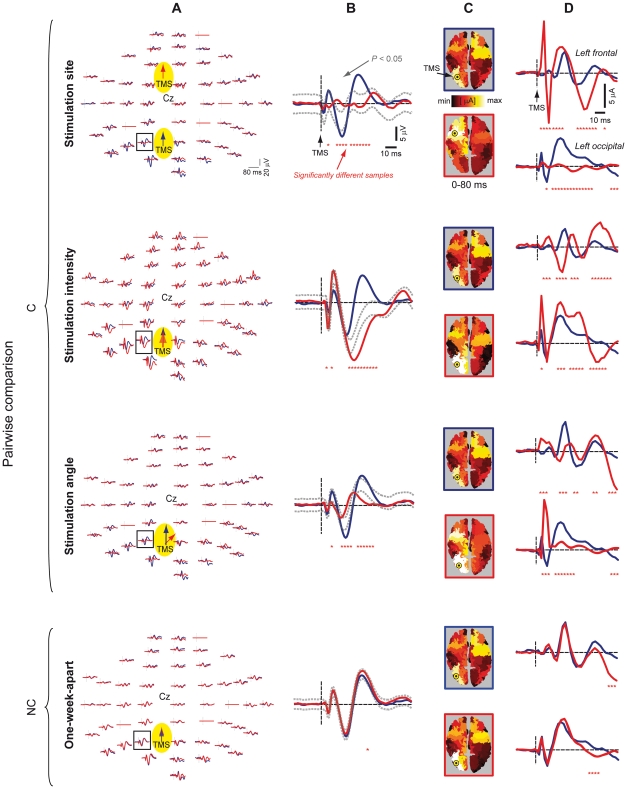
Results of pairwise comparisons between TMS-evoked potentials of a representative subject at the sensor (A,B) and at the source level (C,D). Brain responses to stimulation of BA19 at I% intensity and 0° angle on day1 (blue traces) are compared with brain responses recorded during four different sessions (red traces), during which stimulation parameters were varied one at a time, namely stimulation site (BA6), intensity (I%+10%), angle (45°) and day (day8), resulting in 3 C comparisons and one NC comparison. For each comparison, superimposition of pairs of TMS-evoked potentials in all sensors is displayed in (A), while enlarged view of P1 channel is shown in (B), together with significance boundaries (dotted gray traces) and significantly different samples (red stars). Pairs of TMS-evoked cortical currents are shown in (C) as current density maps and in (D) as temporal profile of current density integrated over the left frontal and left occipital lobules, together with significantly different samples (red stars).

Results of a representative subject are reported in Fig. 2. Here, one particular TMS/hd-EEG session (stimulation of BA19 at I% intensity and 0° angle on day1) is taken as a reference (blue) and compared with four other sessions (red), where stimulation parameters are varied one at a time. Specifically, the site (BA19 vs. BA6), the intensity (I% vs. I%+10%), the angle (0 vs. 45°) and the day (day1 vs. day8) of stimulation were varied, resulting in three C comparisons and one NC comparison. For each comparison, Fig. 2 displays the superimposition of TMS-evoked potentials at the sensor level (A,B) as well as the cortical current density maps (C) and the temporal profile of current density integrated over the left frontal and occipital lobules (D). This representation provides a qualitative description of the overall degree of diversity between different conditions. While TMS-evoked scalp potentials and cortical currents tended to overlap in the NC comparison, they were clearly characterized by divergent spatial-temporal patterns in all the C comparisons, suggesting that the spatial-temporal characteristics of the brain response to a direct perturbation markedly depended on each and every stimulation parameter, e.g. site, intensity and angle.

**Figure 3:**
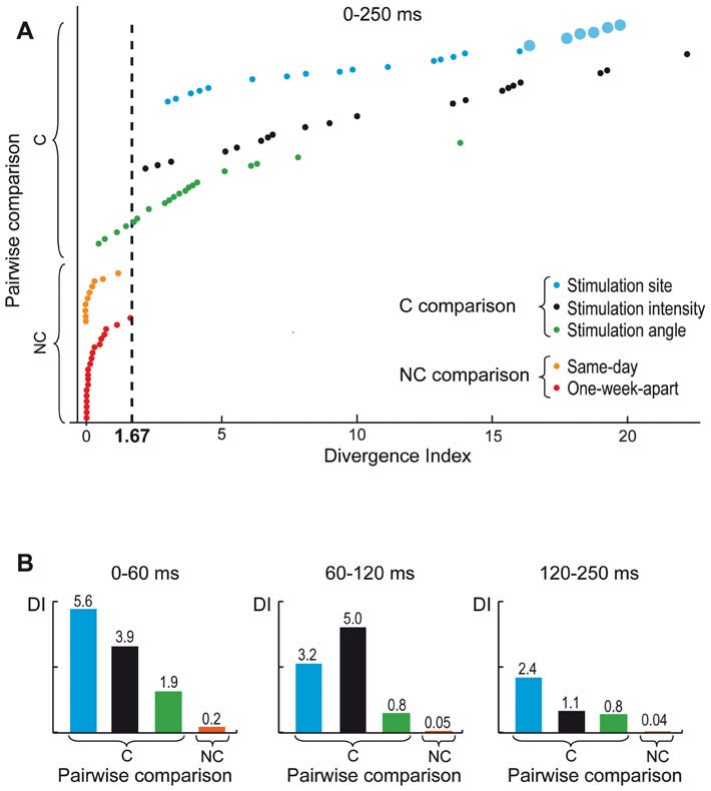
Divergence Index of all pairwise comparisons between TMS-evoked potentials. Single DI values computed over the entire post-stimulus period (250 ms) are shown in (A) with the following color-coding: DIs of the C comparisons for changes in the stimulation site, intensity and angle are represented by cyan, black and green dots, respectively, while DIs of NC comparisons are depicted in yellow for same-day sessions and in red for one-week-apart sessions. DI values computed over different temporal windows of interest (0–60 ms, 60–120 ms, 120–250 ms) are reported in (B) with the same color-coding, except for NC comparisons that are summed together and plotted in orange.

Fig. 3A summarizes the general results obtained from all subjects: each coloured dot represents the DI computed for a specific pairwise comparison. In particular, DIs of the C comparisons for changes in the stimulation site, intensity and angle are represented by cyan, black and green dots, respectively; DIs of NC comparisons are depicted in yellow for same-day sessions and in red for one-week-apart sessions.

### C comparisons resulted in the largest DI values

Generally, comparing the brain responses evoked by TMS pulses delivered over different stimulation sites revealed obvious differences in the space distribution and time course of voltages and currents (Fig. 2, first row). The average DI of all 22 C comparisons between different stimulation sites was 11.45±5.7% (range 3–19.7%). Inspecting single DI values (Fig. 3A) also showed that comparisons between distant areas (i.e. BA6 vs BA19, large cyan dots) was associated with the highest DI values (18.4±1.2%), while pairwise comparisons between nearby sites (i.e. BA6 vs. BA7 and BA7 vs. BA19, small cyan dots) resulted in lower DIs (8.8±4.3%).

Varying stimulation intensity (Fig. 2, second row) resulted in amplitude and latency changes of the main TMS-evoked components, while the general topographical distribution of voltages and currents tended to be preserved. The average DI across the 20 C comparisons between different intensities of stimulation (Fig. 3A, black dots) was 10.88±6% (range 2.31–22.2%).

When changing the angle of the TMS-induced current, the morphology of cortical responses varied in a rather unpredictable way, on a single-case basis (Fig. 2, third row). The average DI value was 3.92±3% (range 0.7–13.8%), with no systematic difference between 0° vs. 45° and 0° vs. 90° pairwise comparisons. In 4 out of 20 angle comparisons, DI values were smaller (Fig. 3A, green dots) than the largest DI obtained comparing experimental sessions with identical stimulation parameters (Fig. 3A, yellow and orange dots).

### NC comparisons results in lowest DI values

When TMS was applied with identical stimulation parameters at different times, the morphology and the spatial-temporal dynamics of TMS-evoked potentials were largely preserved (Fig. 2, fourth row). The average DI was 0.28±0.4% (range 0–1.2%) when comparing same-day recordings and 0.43±0.4% (range 0–1.67%) for pairwise comparisons between one-week-apart sessions. The single DI values for all NC comparisons (Fig. 3A, yellow and orange dots) were always below 1.67%

### Relative differences in the DI value are preserved across latencies

In order to understand whether the observed differences between TMS-evoked potentials were preserved over the entire post-stimulus period, rather than limited to a specific latency, we computed the DI over three subsequent post-stimulus intervals, namely 0–60 ms, 60–120 ms and 120–250 ms (Fig. 3B). For each type of pairwise comparison, the average DI computed at early latencies (0–60 ms) was significantly higher (*P*<0.05) as compared to the one computed for late latencies (120–250 ms), although the relative differences among types of comparison were preserved across all time intervals (*P*<0.01).

### ROC Analysis

**Figure 4:**
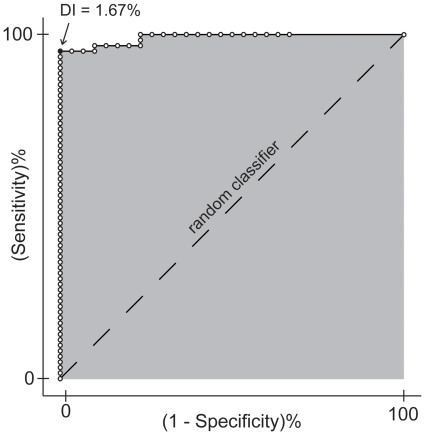
ROC analysis applied to the DI. ROC curve is depicted as a solid black line, interspersed by blank dots representing the values of sensitivity and specificity (repeatability) associated to single DI values. The optimal DI value of 1.67% computed according to the Younden index [34] is shown as a black dot. Dashed line represents the ROC curve of a random classifier. Gray-shaded region indicates the area under the curve.

Results of ROC analysis showed that the optimal DI threshold according to the Younden index was 1.67% and yielded a 95.1% sensitivity and 100% specificity (repeatability), corresponding to an overall accuracy of 96.7%. The efficacy of DI in reliably quantifying the pairwise differences between TMS-evoked potentials was 99.1%, as measured by the area under the curve (Fig. 4).

## Discussion

How accurately TMS-evoked potentials can detect actual changes in cortical responsiveness? If the EEG response to TMS tends to be stereotypical, actual changes in cortical responsiveness may go undetected (low sensitivity). On the other hand, if TMS/hd-EEG measurements tend to be too variable and noisy, changes in cortical responsiveness may be overestimated (low repeatability).

Defining sensitivity and repeatability concerns the interpretation of the responses to any kind of stimulation: however, this task is particularly relevant when interpreting the EEG responses triggered by TMS, a technique that activates the brain in a way that is non-ecological and that is difficult to control.

Unlike sensory stimulation, TMS activates simultaneously a rather large cortical volume containing both inhibitory and excitatory fibers, possibly belonging to different functional subsystems. Thus, it is possible that different TMS perturbations may result in EEG responses that engage many different circuits and that are largely overlapping. In addition, TMS not only perturbs cortical neurons directly but may also activate the brain indirectly, due to the stimulation of scalp nerves and to the click sound associated with the coil's discharge over the subject's head. For this reason, it is also conceivable that differences in the brain's reaction may be partially obliterated by an invariant event-related potential triggered by unwanted somatosensory or/and acoustic stimulations. Altogether, these factors may significantly hamper the sensitivity of TMS-evoked potentials. On the other hand, due to the complexity of the technique, TMS-evoked potentials may also lack repeatability, by showing accidental changes related to stimulation and/or recording errors. Indeed, stimulating directly the cortical surface involves the control of several factors, since a large number of cortical locations can be arbitrarily selected and perturbed, each one with several stimulation parameters (e.g. intensity, pulse waveform, and orientation of the magnetic field). Thus, a lack of precise control of these parameters across subsequent TMS/hd-EEG sessions, may result in large measurement errors and in an apparent modulation of cortical responsiveness. Similarly, other factors, such as EEG sensors positioning, coil temperature, calibration of amplifiers, etc., may, if not adequately controlled for, affect the repeatability of TMS-evoked potentials.

In this work, we used controlled changes in the stimulation parameters (site, intensity, and angle of the induced electric field) and repeated longitudinal measurements (same day and one week apart) in order to jointly evaluate the sensitivity and repeatability of TMS/hd-EEG. In order to synthetically quantify the extent to which pairs of TMS-evoked potentials, recorded in various conditions, differed in their overall spatial-temporal pattern, we employed non-parametric statistics to calculate a Divergence Index (DI). ROC analysis showed that an optimal DI threshold of 1.67%, yielded a 96.7% accuracy (95.1% sensitivity and 100% specificity) of TMS-evoked potentials in detecting whether a change in stimulation parameters occurred, or not. The finding that TMS-evoked potentials, rather than being stereotypical or noisy responses, reflect to a large extent deterministic properties of the stimulated cortical circuits has different implications, as discussed below.

### Sensitivity of TMS-evoked potentials and the differentiation of cortical circuits

Integration and differentiation (or functional specialization) within regions are fundamental organizing principles of thalamocortical networks [35], [36]. While integration refers to the ability of the elements of a system to interact with each other, differentiation may be defined as the system's ability to react in different ways to different perturbations. TMS/hd-EEG, by directly exploring causal interactions (effective connectivity) among cortical areas, may provide a dependable evaluation of thalamocortical integration [16], [37], [38]. On the other hand, to the extent that TMS-evoked potentials are sensitive to changes in perturbation parameters, they may also gauge the degree of differentiation within thalamocortical networks. The present work shows that, at least on a coarse grain, different cortical perturbations result in a degree of response differentiation that is consistently higher compared to random test-retest variability (Fig. 3 and 4).

In the present experiments, changing stimulation parameters almost invariably resulted in higher DIs compared to the no-change conditions (Fig. 3A). Importantly, this finding was not limited to the early latencies, and indeed DI values for the C conditions were significantly larger until 250 ms post-stimulus (Fig. 3B). This evidence demonstrates that the EEG response to TMS is primarily due to direct cortical stimulation and to the ensuing reverberation of activity in a specific network of connected elements. Moreover, if TMS-evoked potentials were heavily contaminated by somatosensory, or auditory event-related potentials, the EEG responses generated when stimulating the head in two sites located a few centimetres away, rotating the stimulation angle, or increasing slightly the intensity of stimulation would have a very similar morphology. Thus, our finding suggests that the collateral stimulation of peripheral nerves by TMS plays a little role in the generation of TMS-evoked potentials.

Changing the site of stimulation resulted in very different responses and in high DI values that were even higher when the responses triggered in areas located far away (area 19 vs. area 6) where compared (Fig. 3A, large cyan dots). This variability in the cortical response reflects specific properties of the stimulated circuits and may be ascribed to local differences in cortical excitability [17], to differences in the frequency tuning of corticothalamic modules [18], [39] and to differences in the pattern of cortico-cortical connectivity [4], [12], [24].

Rotating the coil in the same area produced smaller modifications of TMS-evoked potentials as compared to changes of stimulation site. Indeed, 4 out of the 20 pairwise comparisons between recording sessions with different angles resulted in a DI <1.67%, i.e. the optimal threshold that maximized sensitivity and specificity (repeatability) of TMS-evoked potentials in the ROC analysis. Previous work [40] showed that by stepwise rotating the coil relative to left motor cortex, the largest muscle responses were obtained when the coil was 50° to the parasagittal plane, with the induced current in axis with the main direction of the axons in the motor “hand knob”. In addition, TMS coil orientation has been shown to affect the motor threshold [41], the degree of selectivity when stimulating different peripheral muscles [42] and even cognitive functions [43]. However, while the main orientation of motor cortex axons is quite predictable across subjects, little is known, a priori, about the main orientation of fibers in other brain areas, such as the ones stimulated in this study (BA6, BA7 and BA19). Thus, it is likely that the 4 pairwise comparisons between different stimulation angles resulting in low DI values, may be due to a virtually negligible variation of the induced current direction as compared to cortical axons. In fact, only by integrating TMS/hd-EEG with high resolution structural imaging techniques, such as diffusion tensor imaging (DTI), one may be able to control, in any cortical area, the coil's orientation with respect to the main direction of axons.

Certainly, in order to evaluate the fine grain of cortical differentiation with TMS/hd-EEG, one should design ad hoc experiments where stimulation parameters are varied in a systematic way (i.e. by moving/rotating the coil several times by a constant step). Meanwhile, it would be interesting to test whether the DI resulting from two different perturbations decreases in physiological (sleep, anesthesia) and pathological (coma, epilepsy) conditions, where the capacity for integration and differentiation in thalamocortical circuits is thought to be altered [35], [37], [44], [45].

### Repeatability of TMS-evoked potentials and longitudinal changes in cortical circuits

TMS-evoked potentials recorded on the same day, with the same stimulation parameters (Fig. 3, yellow dots) were very stable (mean DI 0.28%), despite the fact that, between the two measurements, several (from 5 to 8) other sessions of repetitive TMS were carried out. It is well known that repetitive TMS pulses delivered at low (<1 Hz) and high (>5 Hz) stimulation frequency can respectively induce a reduction [46] and an increase [22], [47] of brain excitability [48]. Throughout the experiment, we used a stimulation frequency jittering randomly between 1.1–1.4 Hz. Our results indicate that this particular stimulation rate does not induce significant brain reorganization/plasticity processes and may be used to probe repeatedly the excitability of cortical circuits without significant interference.

The mean DI between TMS-evoked responses recorded during identical experimental sessions performed one week apart (0.43%) was slightly higher than mean DI between same-day sessions (0.28%). This minor discrepancy might be ascribed to different factors: i) small co-registration errors, that clearly reduce the reproducibility of navigation; ii) small errors in re-positioning the EEG cap; iii) unavoidable and unpredictable biological variability due to changes in brain excitability that likely takes place on the time-scale of several days. Nevertheless, this comparison clearly demonstrated that TMS-evoked potentials, besides being sensitive to changes, are also very stable over time. Thus, as suggested by the ROC curve in Fig. 4, repeating after one week a given perturbation and observing a DI >1.67% would strongly indicate that, in the mean time, some change occurred in the brain circuits. In principle, identifying a cut-off level above which one can decide whether a change in brain responsiveness occurred, or not, is crucial if one wants to use TMS/hd-EEG to track over time pathological alterations, plastic changes and therapy-induced modifications in cortical circuits. The DI can be automatically computed at the sensor as well as at the source level (Fig. 2) on a large matrix of spatial-temporal data, without having to select particular peaks, or components. By design, the DI tends to better capture changes in amplitude than changes in shape, since two responses with identical shape but with different amplitude will be maximally different. This feature may represent a limitation or an advantage, depending on the case (see below). In the present experiments, increasing TMS stimulation intensity by 10% produced changes that were reliably detected by the DI. This finding suggests that calculating the DI on repeated TMS/hd-EEG sessions may be effective in revealing rather fine modifications in cortical excitability (indexed by the response's amplitude) due to pathological alterations, i.e. stroke, epilepsy and depression [49]–[52] or therapeutic interventions, i.e. electroconvulsive therapy, rTMS, neurorehabilitation or drug administration [51], [52].

### Limitations of the study

The DI values computed in the C conditions allowed detecting changes in the stimulation parameters with excellent accuracy: however their absolute values spread over a wide range (Fig. 3A). This dispersion may be due to a differential susceptibility of different cortical sites to changes in stimulation parameters. For example, if delivering TMS pulses on a specific cortical region at I% intensity activates locally most of the axons, increasing the stimulation intensity by 10% in this particular area would not dramatically change the electrical response and would result in a low DI. Similarly, as already discussed above, stimulating at different angles would not make much difference if the fibers in the target area are oriented in all directions (anisotropic arrangement). The contribution of these factors to the morphology of TMS-evoked potentials cannot be easily predicted, even if some insights may be provided by the integration of high-resolution structural neuroimaging (such as DTI). At any rate, a systematic understanding may only be achieved by varying parametrically the perturbation parameters, e.g. stimulating at several locations uniformly distributed on the scalp, delivering TMS pulses at progressively higher intensities from threshold to saturation, rotating gradually the angle of the induced current to span the whole circle. Such an extensive mapping of cortical electrophysiology was clearly beyond the scope of the present work, which was primarily aimed at evaluating, technically, the general level of accuracy of TMS/hd-EEG. Therefore, here, we tested only a limited set of all possible stimulation parameters, a task that still required considerable experimental and methodological efforts, e.g. recording and analyzing 100 TMS/hd-EEG sessions and performing 92 pairwise comparisons between TMS-evoked potentials.

Various classifiers, describing differences between evoked potentials, could have been used to build the ROC curve. In this work, we implemented the DI because it allows to quantify the differences between TMS-evoked potentials starting directly from the entire matrix of spatial-temporal data, without requiring a priori information, and to synthesize them into a single number. However, the ability of the DI in detecting modifications of TMS-evoked potentials due to physiological and/or pathological alterations in cortical circuits should be carefully evaluated. Indeed, the DI is rather conservative (it corrects for multiple comparisons in time) and explores the entire post-stimulus period, at all sensors. Thus, since several channels and latency ranges are often not involved by a TMS-evoked potential, one should generally expect low absolute DI values. In fact, we found that maximum DI values were around 20% (Fig. 3). When testing more specific hypotheses, other methods of classification (e.g., restricting the DI in space and time, template-matching, Mahalanobis distance) could be chosen. Clearly, the aim of the present study was not to develop an optimal classifier, but to use the most general one in order evaluate the accuracy of TMS/hd-EEG with a data-driven approach.
